# Diagnosis and Treatment of Metastatic Colon Cancer in Pregnancy First Presenting as Multiple Liver Masses: A Case Report

**DOI:** 10.7759/cureus.53218

**Published:** 2024-01-30

**Authors:** Mengjia Wu, Koji Otsuka, Yoshiaki Furusawa, Isao Otsuka, Tokumasa Suemitsu

**Affiliations:** 1 Obstetrics and Gynecology, Kameda Medical Center, Kamogawa, JPN

**Keywords:** multidisciplinary team, diagnosis, treatment, pregnancy, colorectal cancer

## Abstract

Colorectal cancer (CRC) is the second most common cancer in women in Japan. However, it is uncommon during pregnancy. CRC diagnosis during pregnancy is often complicated and delayed due to the overlapping of symptoms, such as abdominal pain and nausea, with those of pregnancy and the limitations placed on potential diagnostic imaging and testing because of concerns for the fetus. A 39-year-old woman was referred from a local hospital at 32 weeks gestation after persistent right abdominal pain, which prompted an ultrasound that showed multiple liver lesions suggestive of malignancy. A combination of non-contrast computed tomography, non-contrast magnetic resonance imaging, contrast-enhanced ultrasound, and colonoscopy was utilized to make a definitive diagnosis; ultimately, colonoscopy confirmed the diagnosis of colon cancer with liver metastasis. A discussion within a multidisciplinary team led to the decision to deliver at 34 weeks by cesarean section and a left hemicolectomy was performed after delivery. The neonate was admitted to the neonatal intensive care unit due to prematurity but had no other complications. Chemotherapy was promptly initiated, and treatment was continued on an outpatient basis. Diagnostic algorithms for CRC during pregnancy are not yet well-established; however, the prognosis of CRC during pregnancy is poor, and clinicians should not hesitate to perform the necessary testing and consult experts in fields such as neonatology, medical oncology, internal medicine, and gastrointestinal surgery. Early diagnosis and intervention are essential for optimizing outcomes for both the mother and the fetus.

## Introduction

Malignancy is diagnosed in approximately 1 in 1,000 pregnancies and the most common tumor types are breast cancer, hematologic malignancies, and dermatologic malignancies [1]. The risk of colorectal cancer (CRC) during pregnancy is estimated to be less than 0.003%, and its incidence is likely to increase with rising maternal age [2]. In Japan, CRC is the second most common cancer type and the most common cause of cancer-related mortality among women [3]. The five-year survival rate in Japan for stage IV colon and rectal cancers is 19.9% and 14.8%, respectively [4]. To date, two main case series have reviewed and analyzed the outcomes of CRC during pregnancy. In the first study by Kocián et al., which included 41 patients, 40% had stage IV disease. The one-year survival rate was 48.6% and the two-year survival rate decreased to 20.8% [5]. In the second study by Pellino et al., metastatic disease was identified in 48% of the patients. Survival was significantly shorter in patients with colon cancer than in those with rectal cancer (26 vs. 73 months, P = 0.0072) [2].

Early diagnosis of CRC in pregnancy, before it presents in advanced stages, is crucial for improving prognosis; however, it has proven challenging because the presenting symptoms, such as abdominal pain, nausea, and rectal bleeding, can often be associated with regular physiological changes during pregnancy. The complexity of these patients also necessitates a multidisciplinary approach to optimize outcomes. Physicians must consider the risks of various diagnostic and treatment options for the fetus and balance them with the benefits for the mother [6]. Here, we present a rare case of stage IV CRC that initially presented with liver metastasis and was diagnosed in the third trimester. A female infant was delivered via cesarean section without complications, with successful tumor resection to relieve the mother’s obstructive symptoms. The decision-making process, which included imaging modalities for diagnosis and treatment options, involved obstetricians and experts in neonatology, medical oncology, internal medicine, and gastrointestinal surgery.

## Case presentation

A 39-year-old woman (gravida 5, para 1) was referred to our hospital at 32 weeks of gestation with persistent right flank pain. Symptoms first presented at 31 weeks of gestation age and were initially thought to be due to kidney stone formation; however, the pain was unresponsive to the analgesics prescribed by her primary healthcare provider, and she was referred after an abdominal ultrasound revealed multiple hepatic lesions. The pregnancy was uneventful until the referral.

**Figure 1:**
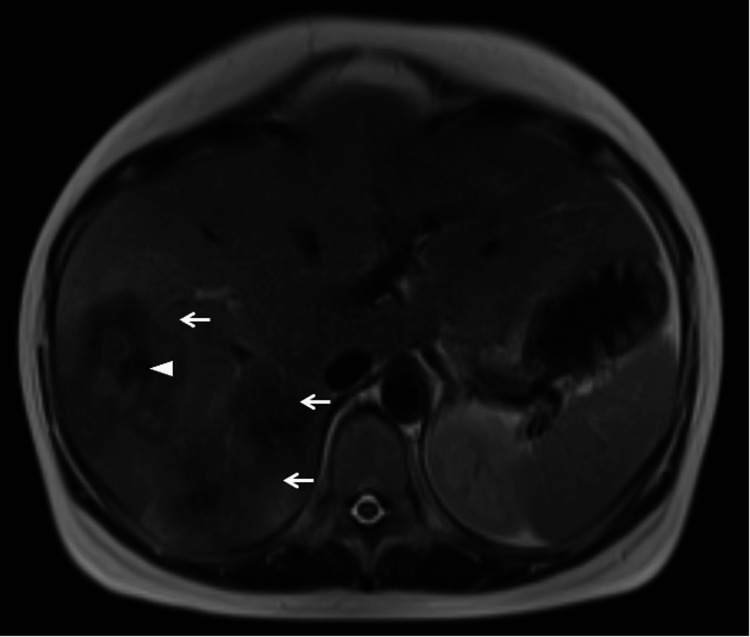
Non-contrast magnetic resonance imaging (MRI; T2-weighted) revealed multiple hepatic lesions (←) with internal necrosis (◁)

**Figure 2:**
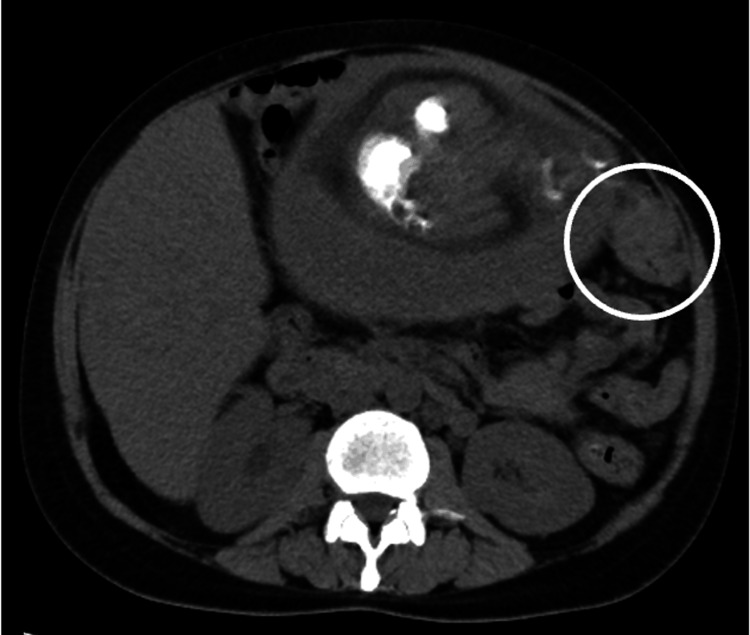
Non-contrast computed tomography (CT) revealed a suspected lesion in the transverse colon (◯)

**Figure 3:**
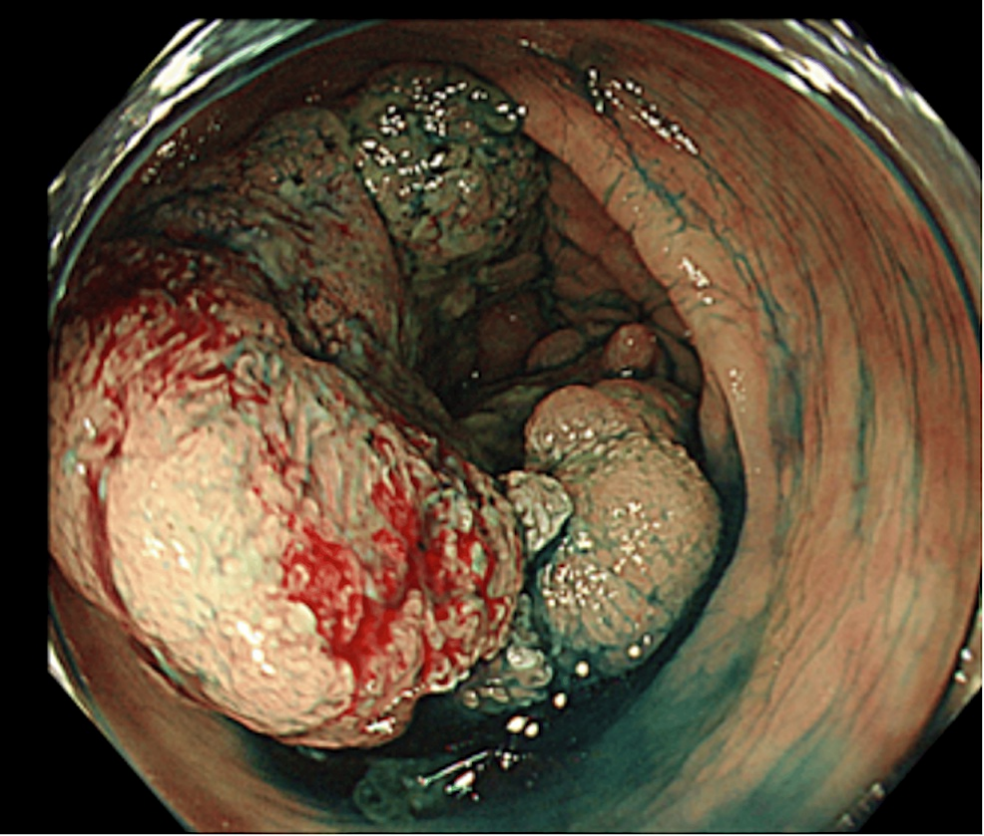
Colonoscopy showed a type 2 invasive tumor in the ascending colon >75% of the luminal circumference

At the time of presentation, vital signs were normal, and physical examination was unremarkable, except for consistent right flank pain. The obstetric examination results were normal. Laboratory results showed moderate anemia (hemoglobin 8.4 g/dL, referred to 7.0-9.9 g/dL as moderate) with normal liver function tests and elevated tumor markers (CEA 51.9 ng/mL (reference range 0-2.5 ng/mL), CA19-9 8080 U/mL (reference range 0-37 U/mL), alpha-fetoprotein 229 ng/mL (reference range for men and non-pregnant women is 0-40 ng/ml but rises throughout pregnancy and can reach 250 ng/mL at 32 weeks of gestation), and PIVKA-II 91 mAU/mL (reference range 0-40 mAU/mL)). The past and current hepatitis infection status was negative. The patient’s mother was diagnosed with renal and bladder cancer in her sixties; however, there is no other family history of cancer. Abdominal ultrasonography revealed multiple masses in the liver, the largest of which measured 6 cm in diameter. Non-contrast magnetic resonance imaging (MRI) suggested malignancy but was equivocal about the possibility of primary or metastatic liver cancer (Figure 1). Non-contrast computed tomography (CT) was negative except for known liver masses. Based on these tests, it was unlikely that her symptoms were due to obstetrical conditions such as hemolysis, elevated liver enzymes, low platelet (HELLP) syndrome, or preterm labor, but were instead due to multiple liver lesions. We considered a liver biopsy; however, the oncology department suggested that given the patient’s age, lack of a family history of primary liver or bile duct cancer, and lack of a medical history of hepatitis, the lesions were more likely to be metastatic. A subsequent review of non-contrast CT images showed a potential lesion in the transverse colon (Figure 2), and contrast-enhanced ultrasound (CEUS) of the liver suggested metastatic liver cancer. Hence, we decided to conduct a colonoscopy first, and if no lesions were found, we would proceed to a more invasive liver biopsy. A colonoscopy confirmed the diagnosis of colon cancer, with a lesion constricting 75% of the colonic lumen (Figure 3). The pathology was consistent with that of well-differentiated tubular adenocarcinoma of the colon. To summarize, the patient was diagnosed with stage IV colon cancer.

**Figure 4:**
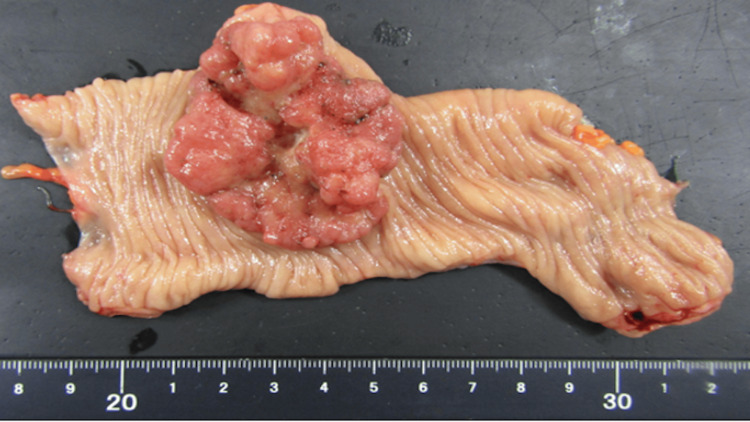
The surgical specimen was moderately differentiated adenocarcinoma. pT3N1MX, tub2, INF a, ly1, v0, Pn1.

After multiple consultations with other experts in neonatology, medical oncology, internal medicine, and gastrointestinal surgery, we prioritized early initiation of chemotherapy. Hence, the decision was made to execute delivery at 34 weeks of gestation after the administration of antenatal corticosteroids. She delivered via planned cesarean section because of a prior cesarean section. During the same operative procedure, a left hemicolectomy was performed immediately after the cesarean section to prevent bowel obstruction during chemotherapy (Figure 4). The neonate was female, with a body weight of 2144 g, an APGAR (Appearance, Pulse, Grimace, Activity, and Respiration) score of 8 (1 min)/9 (5 min), and an umbilical artery pH of 7.382. The neonate was admitted to the neonatal intensive care unit due to prematurity without the requirement of respiratory support and no complications. The infant has no developmental abnormalities as of the two-year follow-up.

The final pathological evaluation was consistent with moderately differentiated adenocarcinoma of the transverse colon and regional lymph node metastasis (positive in 1 out of 12). No placental metastases were observed. The patient was informed in advance of the potential for secondary findings, such as germline mutations, before the comprehensive cancer genome profiling (CGP) test was administered on a biopsy sample of colon cancer. The test revealed a positive v-Raf murine sarcoma viral oncogene homolog B1 (BRAF) mutation but no variants associated with disclosed secondary findings for germline mutations were detected. Based on the results, screening exams were not initiated for her children or siblings after a thorough discussion with the patient and her family.

**Figure 5:**
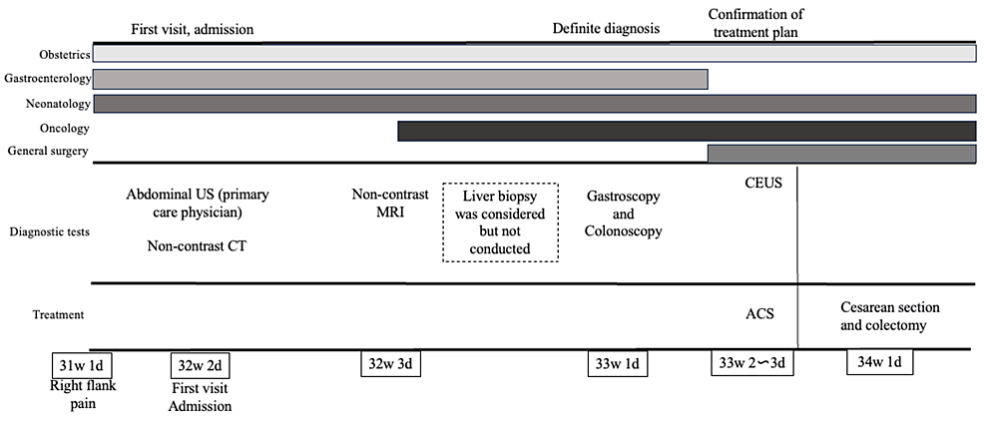
Clinical course showing the involvement of various departments and the tests conducted

The patient had no postoperative complications and chemotherapy (the folinic acid, 5-fluorouracil (5-FU), oxaliplatin, and irinotecan (FOLFOXIRI regimen)) was started on postoperative day 14. She achieved a partial response after approximately 14 months of treatment and underwent liver resection 17 months after the initial treatment. However, a follow-up CT at 21 months revealed new pulmonary lesions suggestive of metastasis; surgical treatment is currently being planned. The clinical course of the patient is summarized in Figure 5.

## Discussion

Herein, we present a rare case of metastatic colon cancer during pregnancy that first presented as multiple liver masses. A multidisciplinary team made decisions regarding diagnostic testing and treatment options. Our patient reported no symptoms other than right flank pain, which could have been easily written off as nonspecific because of pregnancy. Instead, her primary care practitioner promptly investigated with an abdominal ultrasound and was able to make a referral for multiple hepatic lesions within one week of symptom presentation. Despite difficulties in the diagnosis of CRC during pregnancy, multiple departments came together to determine the most suitable diagnostic tests and made an initial diagnosis swiftly within two weeks of presentation. Treatment goals for the patient primarily involved extending survival and improving quality of life, given the advanced stage of cancer. Fetal lung maturation was deemed sufficient for delivery at 34 weeks of gestation after the administration of antenatal corticosteroids and dexamethasone. The decision to perform tumor resection during cesarean section was made after a discussion with oncologists and gastrointestinal surgeons to avoid late complications, such as obstruction, perforation, or bleeding, which can potentially derail chemotherapy.

The diagnosis of CRC during pregnancy is often complicated and delayed because typical symptoms of CRC can be mistaken for common symptoms during pregnancy. Rectal bleeding can be attributed to hemorrhoids, and other symptoms, such as constipation, nausea, vomiting, abdominal pain, and anemia, are nonspecific symptoms that can be attributed to pregnancy [7]. A colonoscopy is the gold standard for confirming the diagnosis of CRC; however, potential complications, including placental abruption due to mechanical pressure on the uterus and maternal hypoxia, have made many clinicians reluctant to obtain a colonoscopy during pregnancy. It can be safely performed under close monitoring during pregnancy when indicated to evaluate conditions such as major lower gastrointestinal (GI) bleeding, suspicion of a colonic mass, or severe diarrhea [8]. Colonoscopy was especially crucial in confirming the histological diagnosis and stricture of the colonic lumen in this case and contributed to the decision to perform colectomy together with cesarean section. On the other hand, ultrasound and non-contrast MRI are generally safe and avoid exposure to ionizing radiation. In our patient, contrast-enhanced ultrasound (CEUS) was used to assess the hepatic masses. There is no official approval for CEUS in pregnancy, as data are still limited. A single-center study of six patients who underwent CEUS to assess hepatic lesions demonstrated no adverse fetal or maternal events [9]. In addition, CT is not commonly used during pregnancy because of concerns regarding its potential radioactive, teratogenic, and carcinogenic effects on the fetus. Pelvimetry exposure can be as high as 50 mGy but is still below the estimated threshold dose for future disability or anomalies and can be conducted if necessary [10].

The two treatment goals are the safe delivery of a viable infant, avoiding complications, and the earliest possible initiation of treatment for patients with cancer. Decision-making is challenging because it is necessary to consider the risks and benefits of potential treatment options for both the mother and fetus. Plans should be individualized and managed by a multidisciplinary team involving obstetricians, gynecologists, oncologists, surgeons, and psychological teams. The treatment plan for colon cancer generally involves surgery and/or chemotherapy; it will depend on factors including the fetus's gestational age, tumor stage, and complicating features in pregnancy or tumor-related symptoms such as bowel obstruction, bleeding, or perforation. If a cesarean section is required for obstetric reasons, as in this case, proceeding with colon cancer resection at the time of cesarean section is usually possible; however, the evidence is limited to individual case studies [11]. Some indications for resection include symptomatic primary tumors with associated perforation and peritonitis, complete bowel obstruction, and severe bleeding. Mild symptoms may improve with the initiation of systemic treatment. However, anastomotic leak post-surgery can lead to death or a possible delay in the initiation of chemotherapy; hence, deciding on resection in cases of mild symptoms is not as straightforward [12]. Chemotherapy is another treatment option for metastatic CRC. FOLFOXIRI is a regime commonly used for treating metastatic CRC. Numerous cases of human 5-FU exposure in pregnancy exist, with most cases occurring in breast cancer treatment [13]. The overall rate of major malformations when the FOLFOXIRI regimen was administered in the second or third trimester was not significantly different from that in the general population [14]. Chemotherapy was not administered because she was close to 35 weeks of gestation, and chemotherapy is usually only continued until 35 weeks of gestation or 3 weeks before the expected due date to avoid the risk of chemotherapy-related complications, such as bone marrow suppression and hemorrhage, during delivery. In this case, since our patient was already scheduled for delivery via cesarean section, the decision was made for resection, even though her symptoms were mild and were relieved after she was started on laxatives because the primary tumor obstructed >75% of the colon luminal circumference and could potentially lead to bowel obstruction and disruption of future treatment. The patient experienced no postoperative anastomotic leak and continued chemotherapy smoothly without any bowel obstruction symptoms.

Evidence for the diagnosis and treatment of colon cancer in pregnancy has largely been limited to individual case reports and case series. Therefore, there is a lack of data to draw definitive conclusions on the risks and benefits of various diagnostic and treatment options. Treatment plans must be individualized for each patient and require the collaboration of experts from various fields.

## Conclusions

CRC is rare and poses a unique challenge to physicians during pregnancy. We present a case of stage IV CRC with liver metastasis during pregnancy that initially presented with right flank pain. By proactively performing CT, colonoscopy, and CEUS, we confirmed the diagnosis before performing cesarean section and colectomy. While exposure to the fetus must be considered, clinicians should not hesitate to perform the necessary diagnostic imaging tests, as diagnosis before presentation in advanced stages is vital for improving patient prognosis. Such decisions regarding imaging choices and subsequent treatment should be made by a multidisciplinary team to optimize care for the mother and fetus.

## References

[1] Van Calsteren K, Heyns L, De Smet F (2010). Cancer during pregnancy: an analysis of 215 patients emphasizing the obstetrical and the neonatal outcomes. J Clin Oncol.

[2] Pellino G, Simillis C, Kontovounisios C (2017). Colorectal cancer diagnosed during pregnancy: systematic review and treatment pathways. Eur J Gastroenterol Hepatol.

[3] (2023). Colon. National Cancer Research Center Cancer Statistics (In Japanese). https://ganjoho.jp/reg_stat/statistics/stat/cancer/67_colorectal.html.

[4] (2023). Regarding the staging of colorectal cancer. National Cancer Research Center Cancer Statistics (In Japanese). http://www.ncc.go.jp/jp/ncch/clinic/colorectal_surgery/160/index.html.

[5] Kocián P, de Haan J, Cardonick EH (2019). Management and outcome of colorectal cancer during pregnancy: report of 41 cases. Acta Chir Belg.

[6] Saif MW (2005). Management of colorectal cancer in pregnancy: a multimodality approach. Clin Colorectal Cancer.

[7] Xu Y, Kong B, Shen K (2018). Adenocarcinoma of the ascending colon in a 31-year-old pregnant woman. A case report. Medicine (Baltimore).

[8] Savas N (2014). Gastrointestinal endoscopy in pregnancy. World J Gastroenterol.

[9] Geyer T, Rübenthaler J, Froelich MF, Sabel L, Marschner C, Schwarze V, Clevert DA (2020). Contrast-enhanced ultrasound for assessing abdominal conditions in pregnancy. Medicina (Kaunas).

[10] American College of Obstetricians and Gynecologists’ Committee on Obstetric Practice (2017). Committee opinion no. 723: guidelines for diagnostic imaging during pregnancy and lactation. Obstet Gynecol.

[11] Walsh C, Fazio VW (1998). Cancer of the colon, rectum, and anus during pregnancy. Gastroenterol Clin North Am.

[12] Feo L, Polcino M, Nash GM (2017). Resection of the primary tumor in stage IV colorectal cancer: when is it necessary?. Surg Clin North Am.

[13] Rogers JE, Dasari A, Eng C (2016). The treatment of colorectal cancer during pregnancy: cytotoxic chemotherapy and targeted therapy challenges. Oncologist.

[14] Kozai L, Benavente K, Obeidat A, Acoba J (2022). FOLFOXIRI in pregnant women with colorectal cancer: a case report and review of the literature. Case Rep Oncol.

